# Syntax errors do not disrupt acoustic communication in the common cuckoo

**DOI:** 10.1038/s41598-022-05661-6

**Published:** 2022-01-28

**Authors:** Csaba Moskát, Márk E. Hauber

**Affiliations:** 1 MTA-ELTE-MTM Ecology Research Group, a Joint Research Group of the Hungarian Academy of Sciences, The Biological Institute, Eötvös Loránd University, Pázmány P. st. 1/C, Budapest, 1117 Hungary; 2 Hungarian Natural History Museum, Baross u. 13., Budapest, 1088 Hungary; 3 Department of Evolution, Ecology, and Behavior, School of Integrative Biology, University of Illinois at Urbana-Champaign, 505 S. Goodwin Avenue, Urbana, IL 61801 USA

**Keywords:** Behavioural ecology, Animal behaviour

## Abstract

When acoustic communication signals are distorted, receivers may misunderstand the signal, rendering it ineffective. Common cuckoos (*Cuculus canorus*) are popularly known for the males’ simple, two-note advertisement calls, the “cu-coo” used for declaring the male’s breeding territories. Cuckoos do not learn their calls (vocal non-learners), so they are expected to have a limited ability to produce different acoustic signals. Nevertheless, male cuckoos appear to make syntax errors (e.g., repeated, reversed, or fragmented elements) even in their simple advertisement calls. We conducted a playback experiment with male cuckoos, broadcasting ten call types, including seven modified calls with errors (e.g. “cu-cu”, and “coo-cu”) and three natural calls used for comparisons (“cu-coo”, “cu-cu-coo”, and interspecific control). Male cuckoos responded in a manner suggesting that the presence of the first (“cu”) note of the natural 2-note “cu-coo” call in any form or combination yield effective signals. However, through the elevated frequency (by about 200 Hz) and greater speed of the “cu” note, the natural 3-note version “cu-cu-coo” call appears to have gained a novel communicative function in signalling with female cuckoos. Thus, syntax errors in calls with the “cu” element are not responsible for changing the function of the male cuckoos’ “cu-coo” call.

## Introduction

Auditory signals play important roles in the communication systems of diverse animal lineages, from arthropods to vertebrates^1^. Acoustic signals are typically degraded during transmission across distances, in ambient noise, and throughout varied habitat structures. For these reasons, not all receivers hear and process the degraded signals properly, and so the production of error-free signals still remains the first step in effective acoustic communication^2^. Songbirds possess a wide variety of acoustic signals, including the ability to develop their repertoire using imitative learning^3^. The resulting birdsongs are typically complex, can contain up to several dozens or hundreds of elements, and have been the subject of extensive prior studies^4^. In contrast, studying bird species with simple, non-learned call repertoires might help to extend our understanding of the basic mechanisms of acoustic signalling. Repeated call sequences may indicate male quality for potential mating partners, as was shown in tawny owls (*Strix aluco*^5^) and European hoopoes (*Upupa epops*^6^). Either lower or higher frequency modulation of male calls or songs might also be perceived as more attractive by female birds, depending on the non-oscine species (reviewed in^7^). When uttering advertisement calls, territory owners may send signals regarding their individual quality for concurrent conspecifics. For example, Eurasian collared doves (*Streptopelia decaocto*) modulate the frequency of the starting element in calls, and male body weight negatively correlates with the proportion of calls lacking the third element^8^. However, signals are not always honest. For example, the scops owls (*Otus scops)* can modify territorial advertisement signals and resident males give a more cautious response to playback of hoots mimicking heavier intruders^9^. This way signallers could send information to receivers regarding their own increased competitive abilities. The structure of sexual and territorial signals can reflect interindividual differences in quality and typically has evolved through male-male competition^10^.

The bird songs and calls can contain several different acoustic elements, but in non-random orders, following the so-called compositional syntactic rules of the focal species and/or population(s)^11–13^. When the sequence of the elements is atypical, syntactic changes may cause either reduced responses, as in field sparrows (*Spizella pusilla*^14^), and winter wrens (*Troglydytes hiemalis*^15^), or provoke more aggressive responses from receivers, as in Eurasian skylarks (*Alauda arvensis*^16^), while sometimes no difference is elicited (e.g. in indigo buntings, *Passerina cyanea*^17^). Syntactic changes to typical acoustic elements also reduce the information content of the signal in the California thrasher (*Toxostoma redivivum*), where the structural order of elements advertises territorial defence^18,19^. In the Japanese tit (*Parus minor*) syntactic changes in syllable order cause semantic changes^13,20^. Another example is the Bonelli’s warbler (*Phylloscopus bonellii*), a species with a very simple song (a trill is repeated about ten times), where syntactic changes in the structure of the acoustic elements (e.g. reversion, inversion, frequency modulation, etc.) significantly reduce receivers’ responses^21^.

**Figure 1:**
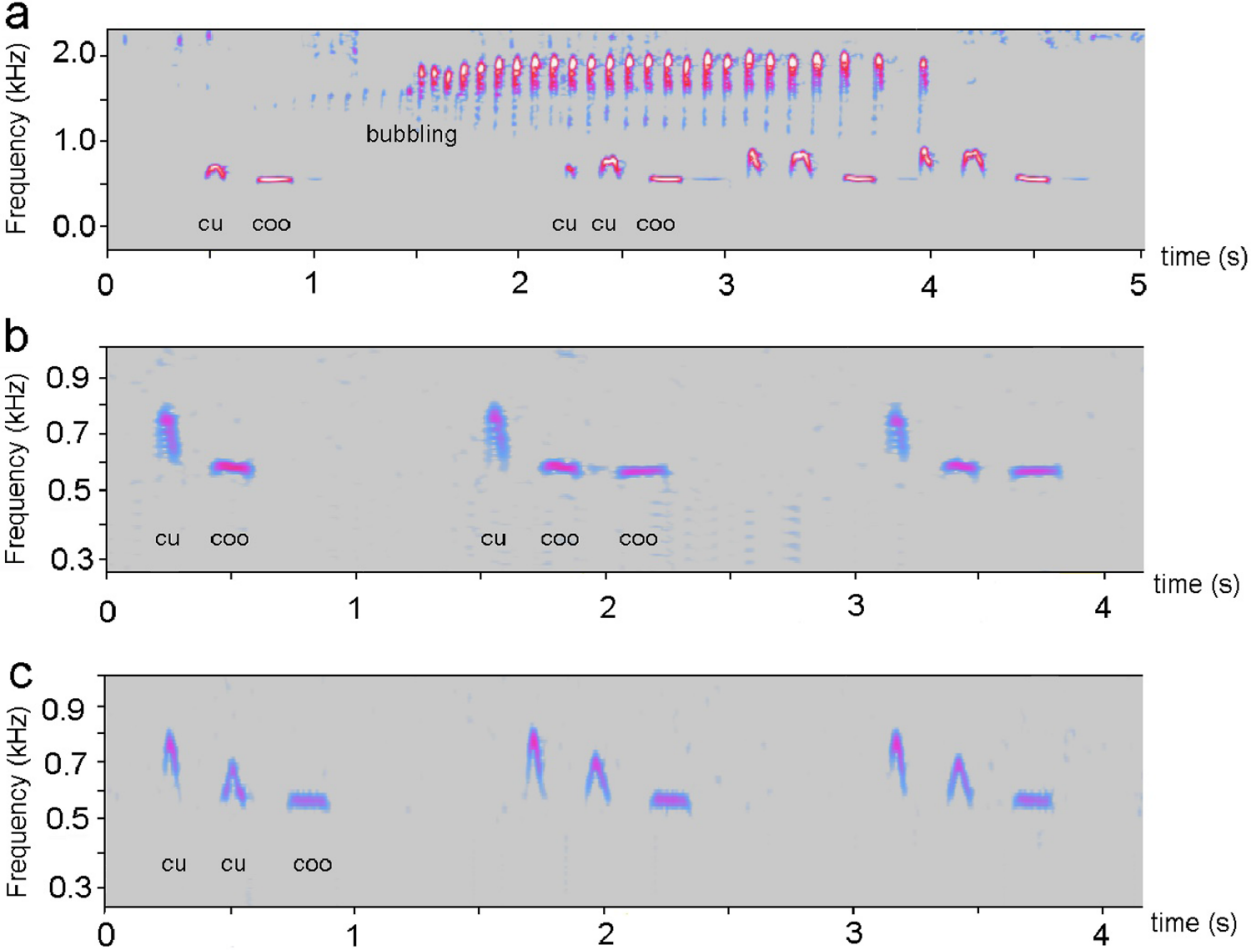
Examples of male (and female) common cuckoo calls. **(a)** The natural forms of 2-note (“cu-coo”) and 3-note (“cu-cu-coo”) calls of males, together with a female-specific bubbling call also indicated on the sonogram. **(b)** A “cu-coo” call and the first two calls in a series of “cu-coo” calls with a syntactic error, where the second note is repeated (the “coo” element, i.e. ”cu-coo-coo”). **(c)** A normal “cu-coo” call and calls from a series of “cu-cu-coo” syntax errors. A proportion of **(b,c)** were uttered by the same individual cuckoo. Note that the natural 3-note call in **(a)** is normal (natural), but the 3-note call in **(c)** differs from it in several respect (see more details in the text): it specifically suffers from a syntactic error, where the first note of the normal “cu-coo” call (“cu”) is repeated. In **(c)** the decreasing frequencies of the first two “cu” elements are a unique case, showing that cuckoos are capable of frequency modulation (typically, the second note is about as high as that of first element).

Apart from songbirds (oscine Passeriformes), hummingbirds (Trochiliformes), and parrots (Psittaciformes), most other bird lineages have relatively simple and innate abilities for acoustic communication. These lineages together are called vocal non-learners^22,23^ and typically have a simple vocal repertoire; however, a simple repertoire does not necessarily mean poor acoustic abilities^24–26^. One of the best examples for an avian species with simple call repertoire is the common cuckoo (*Cuculus canorus*), an obligate brood parasitic species with its famous “cu-coo” call^27^, which is the main call type of adult males^28^. The call contains 2 elements (notes), with low frequencies (400–900 Hz) and they are separated by silence (Fig. 1a). The males utter this call more or less “continuously” in their breeding season, and it can be heard by people at long distances (~ 2–3 km)^29^. Its function as an advertisement type of call in the males’ territorial behaviour^28^ was supported by playback studies^30,31^. However, presently we do not know whether the “cu-coo” call can advertise male quality or not. It was hypothesized that males in better quality may produce longer call sequences than do lower quality individuals^29^. Consequently, a playback experiment generated less intense responses by territorial males to drastically shortened call sequences (reduced to 20%^32^). However, more recently, males’ calling rates showed no statistical relationship with the body weight of male cuckoos^33^. Male common cuckoos also did not use their 2-note advertisement call for mate attraction, and instead uttered a variant of this call (the 3-note “cu-cu-coo”^34,35^) in male–female communication contexts. Additionally, an aberrant but still communicatively effective version of the basic “cu-coo” call (e.g., “cu-kee”) has also been reported in the literature^29,36^. However, aberrant calls differ from compositional syntax errors, as the structures within the notes are modified, which are represented by the altered shapes of their spectrograms (see e.g. Fig. 1 in^36^).

In our study we tested if changes in the compositional sequence of acoustic elements (i.e., syntax errors) reduce responses of male common cuckoos to conspecific playbacks. As common cuckoos utter their simple “cu-coo” calls in long series continuously, we hypothesized that the ordering of the two elements and its variants in individual calls would have less relevance than the type of the element. As a previous study^36^ already revealed that the first element (“cu”) elicited similar response in receivers as the complete “cu-coo” call, we predicted that the first element (“cu”) would have more information content for male cuckoos’ acoustic communication than the “coo” element. We also predicted that the lack of this element would make the call ineffective for receivers. Finally, we predicted that male cuckoos would respond with less intensity to any form of the 3-note cuckoo calls (“cu-cu-coo”) as the natural form of the 3-note calls has a different intraspecific function, i.e. pair-bonding with female cuckoos^34,35^.

## Results

**Figure 2:**
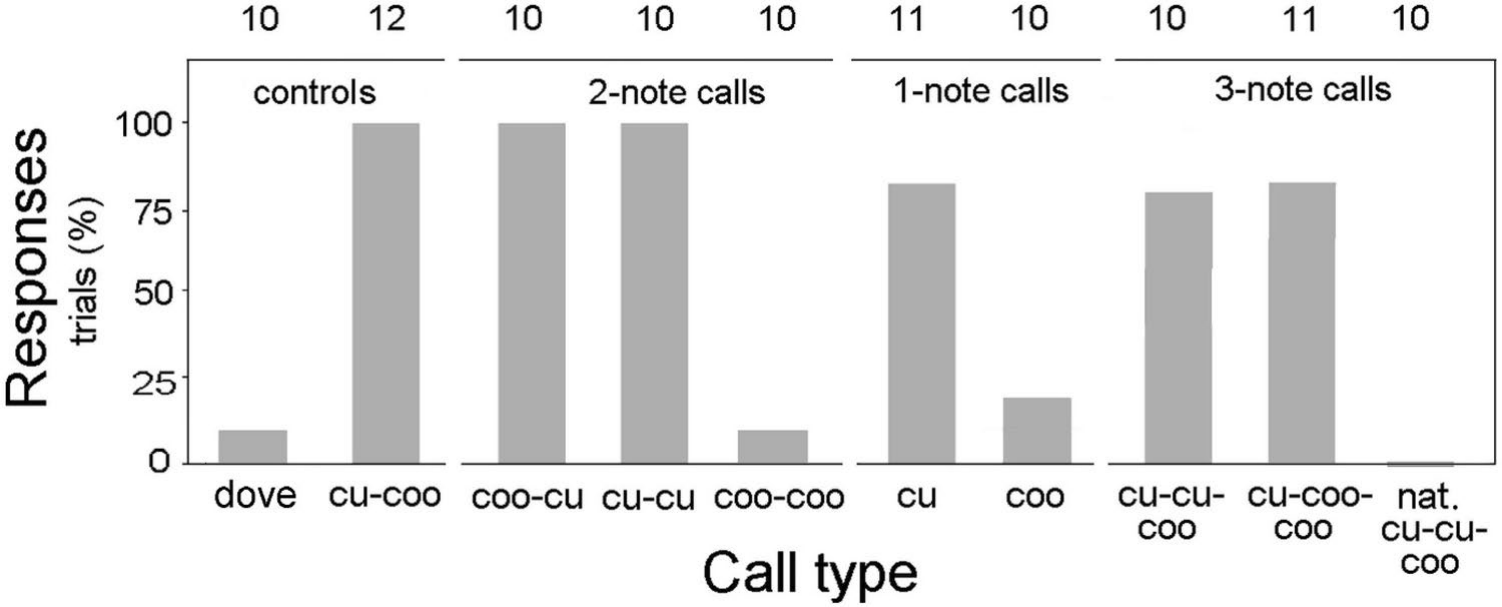
Male common cuckoos’ approach to the speaker during playback with different call types (Y/N). Numbers above bars indicate sample sizes.

Altogether we conducted 104 experimental playback trials, including the natural control (“cu-coo”), interspecific control (dove calls), and the 3-note natural (“nat. cu-cu-coo”) calls. Male cuckoos responded significantly more (in 80–100% of trials) to all the types of playback stimuli when these included the “cu” element, but they did so much more rarely (in 0–20%), when the call contained the “coo” element only (Fig. 2). Both of our fixed effect linear models, either with the binary response variable (response: yes or no) or with continuous response variable (closest distance in m) revealed that responsiveness did not differ in the one-, two-, and three-note calls (Table 1). The starting position of the focal cuckoo, i.e. the distance between the speaker and the bird (“starting distance”; m), did not affect the response to playback in the first model (P = 0.912), but was significant in the second model (P < 0.001; Table 1). This means that locality of the focal cuckoo at the start of the playback trial did not affect cuckoos’ response to playback, but affected the intensity of their response (e.g., approaching the loudspeaker). Playback types effects were similar in the two analyses, the playbacks with a “cu” note (“cu-coo”, “cu”, “cu-cu”, “coo-cu”, “cu-cu-coo”, and “cu-coo-coo”) differed significantly from the dove control used for reference (Table 1). Playbacks with no “cu” note (“coo”, and “coo-coo”), as well as the natural 3-note call, which has a divergent function (see below) elicited similar responses to the dove control playbacks. Starting distance also did not affect closest distance (P = 0.883) when the three 3-note calls were analysed separately by logistic regression (Table 2).

**Table 1:** Parameter estimates of fixed effect linear models of male common cuckoos’ responses to playbacks.

(a) Dependent variable: response					(b) Dependent variable: closest distance			
Parameter	Estimate ± SE	df	t	P	Estimate ± SE	df	t	P
“cu-coo”	0.899 ± 0.128	93	7.031	< 0.001	−32.013 ± 4.484	93	−7.139	< 0.001
“cu”	0.716 ± 0.132	93	5.437	< 0.001	−28.602 ± 4.618	93	−6.194	< 0.001
“coo”	0.099 ± 0.134	93	0.738	0.463	−7.233 ± 4.693	93	−1.541	0.127
“coo-cu”	0.896 ± 0.139	93	6.461	< 0.001	−25.927 ± 4.861	93	−5.334	< 0.001
“cu-cu”	0.896 ± 0.137	93	6.532	< 0.001	−24.133 ± 4.812	93	−5.016	< 0.001
“coo-coo”	−0.004 ± 0.138	93	−0.027	0.978	−8.254 ± 4.825	93	−1.711	0.090
“cu-cu-coo”	0.696 ± 0.138	93	5.063	< 0.001	−25.510 ± 4.822	93	−5.290	< 0.001
“cu-coo-coo”	0.716 ± 0.131	93	5.448	< 0.001	−22.907 ± 4.610	93	−4.969	< 0.001
“nat. cu-cu-coo”	−0.102 ± 0.135	93	-0.758	0.450	−4.003 ± 4.719	93	−0.848	0.398
Dove	0 ± 0	–	–	–	0 ± 0	–	–	–
Starting distance	−0.0003 ± 0.003	93	−0.111	0.912	0.559 ± 0.108	93	5.177	< 0.001
Intercept	0.114 ± 0.156	93	0.729	0.468	19.271 ± 5.481	93	3.516	0.001
Model summary	Type III tests of fixed effects: Intercept: F = 30.576, df = 1,93, P < 0.001 Playback types: F = 20.621, df = 9,93, P < 0.001 Starting distance: F = 0.12, df = 1,93, P = 0.912				Type III tests of fixed effects: Intercept: F = 0.140, df = 1,93, P = 0.709 Playback types: F = 12.760, df = 9,93, P < 0.001 Starting distance: F = 26.803, df = 1,93, P < 0.001			

(a) Dependent variable: response (Y/N, where yes: approached the speaker, no: did not approach the speaker). Independent variables: playback type, as a categorial variable (categories: “co-coo”, “cu”, “coo”, “coo-cu”, “cu-cu”, “coo-coo”, “cu-cu-coo”, “cu-coo-coo”, “nat. cu-cu-coo” and interspecific control = dove), and starting distance (m), as a covariate (the distance of the focal bird to the speaker at the beginning of the playback trial). (b) Dependent variable: closest distance (m). Independent variables: playback types, with starting distance (m) as a covariate.

**Table 2:** Binary logistic linear model of male common cuckoos’ responses (Y/N; dependent variable) with the independent variables of 3-note playbacks (“cu-cu-coo” and “cu-coo-coo”), relative to natural 3-note cuckoo calls (“nat. cu-cu-coo”; reference category) and starting distance (m).

Parameter	B ± SE	Wald χ^2^	df	P
Playback types		8.485	2	0.014
“cu-cu-coo”	3.233 ± 1.260	6.587	1	0.010
“cu-coo-coo”	3.271 ± 1.219	7.204	1	0.007
Starting distance (m)	0.007 ± 0.045	0.022	1	0.883
Intercept	−2.231 ± 1.877	1.413	1	0.235
Model summary	Nagelkerke R^2^ = 0.627; Hosmer-Lemesow test: χ^2^ = 5.655, df = 8, P = 0.686			

**Figure 3:**
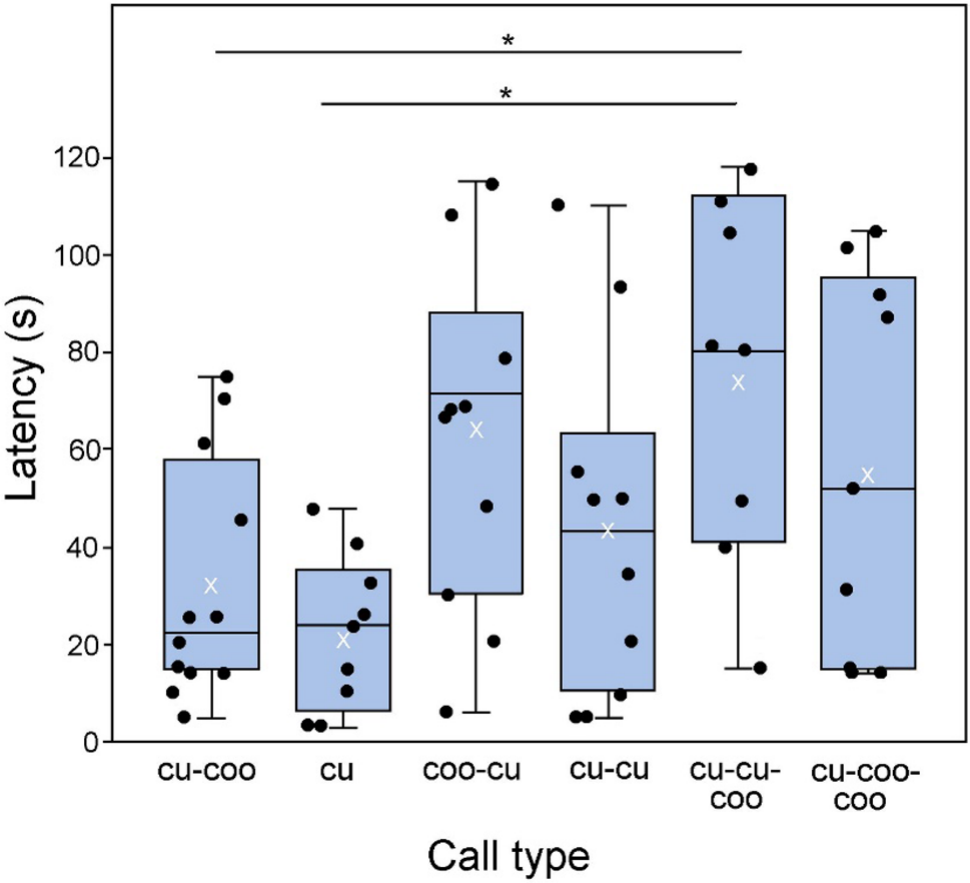
The latency of male common cuckoos’ responses to playbacks with different call types containing the “cu” element. (The trial categories of “coo”, “coo-coo”, “natural cu-cu-coo” and “dove control” are not shown here as the consequence of almost no responses to the playback, i.e. 0 ≤ n ≤ 2.) The box plots show the median (central horizontal line), mean (white x), 75th and 25th percentiles (top and bottom of the box), and the maximum and minimum values (top and bottom whisker), with the jittered points representing each data value. Asterisks indicate significant relationships calculated by Tukey post hoc tests following an ANOVA (*P ≤ 0.05).

Latency to respond was longer to the 3-note calls than to the 2-note control (“cu-coo”) (Fig. 3). The ANOVA of latencies revealed significant difference among the call types (F_5,52_ = 3.558, P = 0.008). Our observation was that the latency of response increased when the complexity of the signal was increased, and when the played call was more different than the natural 2-note “cu-coo” call (Fig. 3). Tukey’s post hoc tests of among the groups in the ANOVA revealed that a significant difference was found between the “cu” and the experimental “cu-cu-coo” calls (mean difference = 52.76, SD = 15.540, P = 0.02). Furthermore, a significant difference was also seen between the “cu-coo” and experimental “’cu-cu-coo” calls (mean difference = 42.96, SD = 14.597, P = 0.05).

It is plausible that the natural 3-note call (“nat. cu-cu-coo”) develops or otherwise originates from the natural 2-note (“cu-coo”) call. Interestingly, a logistic regression revealed that male cuckoos responded differently (more often) to both of our experimentally constructed 3-note calls (“cu-cu-coo”, and its “cu-coo-coo” variant) than to the natural 3-note (“nat. cu-cu-coo”) calls (much more rarely; playback type: Wald χ^2^ = 8.484, df = 2, P = 0.014; Fig. 2). The starting distance of the experimental trial did not affect cuckoo responses (Wald χ^2^ = 0.022, df = 1, P = 0.883; see for details of model summary and parameter estimates in Table 2).

## Discussion

Our playback experiment with different combinations of the elements of the “cu-coo” calls revealed that the “cu” note has more signalling value in male cuckoos’ communication than the “coo” note. In 2-note calls both “cu-cu” and “coo-cu” were as highly effective (100%) as the typical call (“cu-coo”), whereas focal males almost never (10%, i.e. 1 out of 10 cases) responded to the “coo-coo” calls. The “cu” notes also received more responses from cuckoos than the “coo” notes when only one of the elements of the call was played back (“cu” 82% vs. “coo” 20%). Among the 3-note calls all playback call types contained the “cu” element (“cu-cu-coo”, and “cu-coo-coo”) and both 3-note call types were similarly effective.

**Figure 4:**
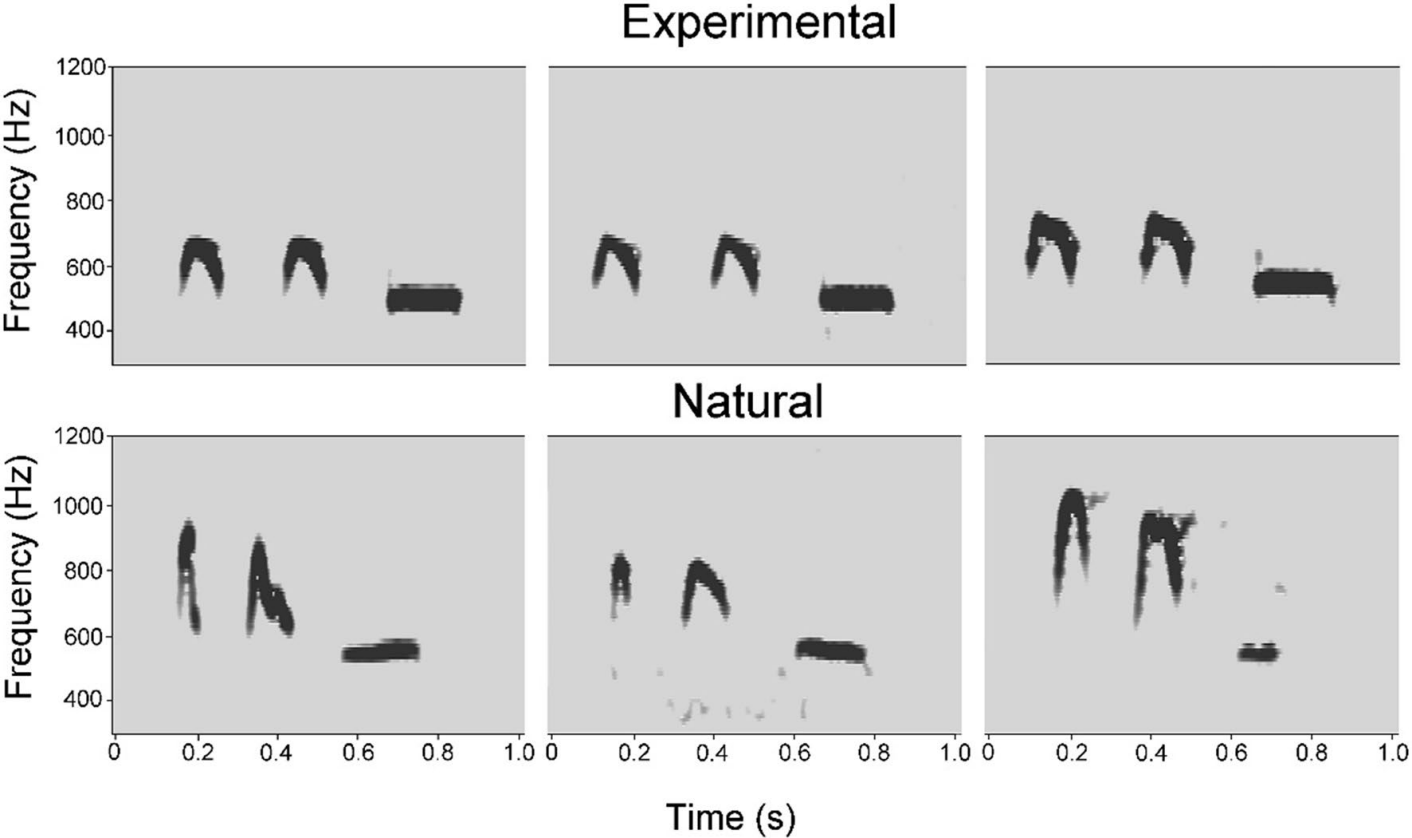
Examples of the 3-note “cu-cu-coo” calls used for playbacks. Top line: experimental 3-note calls, generated from 2-note “cu-coo” calls by repeating the first note. Bottom line: the faster and higher frequency natural 3-note calls. All spectrograms were generated from calls of different individual cuckoos.

Syntax changes may affect semantics in animal communication, with the extreme case seen in primates where semantic changes evolved to a language-type communication means^37^. However, in the common cuckoo the unusual structural composition in some calls can be regarded as simple syntax errors made by the signallers, and these do not lead to any semantic (communicative functional) change. The seemingly singular exception, the 3-note natural “cu-cu-coo” call, has an altogether different function. In turn, the natural 3-note variant of the 2-note cuckoo call is used by males for duetting with females^35^. However, this natural 3-note variant is uttered at somewhat higher frequency, elevated by about 200 Hz, and with higher speed (less pause among notes) than our experimental versions of the same (Table 3, see details in “Methods”; Fig. 4). These changes in frequency and speed seem to be important, as the natural 3-note calls were significantly less effective than our 3-note variants to generate response strength from conspecific males. We conclude that the simple repeat of the second note (“coo”) was not sufficient to alter the function of the basic call type. Such response difference between the natural 3-note call and our experimental 3-note call suggests that simple syntax change in the call structure could be stabilized with secondary changes (frequency and speed) naturally to possess a new function. Accordingly, the simple syntactic change (error) does not eliminate original function of the call. Another alternative is that the typical call (“cu-coo”) is uttered in long sequences in natural cases, while the natural 3-note call (“nat. cu-cu-coo”) typically occurs alone or the whole call is repeated at most once or twice^35^. Finally, a study on the Eurasian collared dove also revealed, that uttering 2-note calls instead of the 3-note calls did not cause semantic changes, except when number of notes covaried together with the length of the call^10^.

**Table 3:** Comparisons of acoustic parameters of experimental and natural 3-note (“cu-cu-coo”) calls by one-way ANOVA.

Acoustic parameter	Natural cu-cu-coo	Experimental cu-cu-coo	F	df	p
Length of call (sec)	0.626 ± 0.041	0.761 ± 0.025	79.258	1,18	< 0.001
Minimum frequency (Hz)	469.5 ± 41.377	464 ± 16.633	0.159	1,18	0.695
Maximum frequency (Hz)	916.5 ± 80.693	714.2 ± 22.700	58.243	1,18	< 0.001

Mean values of call length (s), minimum frequency (Hz), and maximum frequency (Hz) is shown, together with standard deviation (± SD). This experimental call was constructed by repeating the first element in normal “cu-coo” calls, whilst the natural variant was not manipulated regarding syntax.

In the present study we found that one of the individual notes (“cu”) was responsible for most responses by territory-holder male cuckoos. This suggest that cuckoos use categorial perception when receiving acoustic signals (sensu^38^). However, this cannot exclude the role and contribution of other recognition mechanisms. For example, the second element (“coo”) seems to be superfluous for cuckoos’ territorial signalling, but it may also have other functions. For example, it may help individual or familiarity recognition in cuckoos^31^, as both notes are necessary to discriminate between strangers and neighbours. In songbirds, the sequential order of different syllables may indicate individual-specific characteristics of the signaller (e.g.,^39^), or mobbing behaviour in flocks^40^, but it is not a dependable way in the common cuckoo, a species with a very simple repertoire. Some modulation of frequency and speed could be expected even in cuckoos, but this modulation might lead to changes in signal function, as we saw it in the three-note calls in the common cuckoo (see above; Table 3).

Cuckoos have strictly limited ability to develop new acoustic signals from their set of innate elements. Most of their alternative calls are the variants of the “cu-coo” call with syntax errors. Almost all of the call types we studied, which suffer from syntax error of normal “cu-coo” call, were also observed in the field uttered by free-living cuckoos. However, other variants also could exist. For example, a male cuckoo uttered “cu-coo-cu” at Chernobyl, and once the “coo-cu-coo” variant was also observed^29^. We suppose that these syntax errors are related to the extent of the signal-expression ability of cuckoos, allowing to introduce limited numbers of variations in the sequence and repeat of the elements. Accordingly, the atypical “cu-kee” form^36^ can be regarded as an aberrant form of the second note (i.e., not a compositional syntax error), which we found in several individuals.

Some of our error-like calls are found naturally, but rarely, in common cuckoos, can also be heard in different cuckoo species in the *Cuculus* genus. For example, the African cuckoo (*Cuculus gularis*) has “coo-coo”-type calls^41,42^. Other species may repeat their calls in a varying number of times as, for example, the Himalayan cuckoo (*Cuculus saturatus*) often repeats its “hoop” more than two times, whereas the closely-related Oriental cuckoo (*Cuculus optatus*) utters it two times, only^43^. A comparison based on acoustic parameters of the calls of 67 species in the sub-family Cuculinae revealed higher call similarity in parasitic than non-parasitic species. Parasitic cuckoos tended to have simple and low-frequency calls, but a harmonic structure was more frequent in the non-parasitic group of species^44^.

We conclude that the two elements of the males’ “cu-coo” call have different effectiveness in cuckoos’ acoustic communication. Although in our playbacks we detected only minor behavioural response effects of syntax errors in “cu-coo” calls when such erroneous calls were played back in homogeneous sequences, in nature these errors can be observed rarely. Typically, only one syllable contains error in a longer sequence of “cu-coo” calls, or this error is repeated a few times, although we rarely found individuals when calls with syntax errors where the frequency of the strange call variant exceeded 50% (pers. obs.). We did observe an exceptional case when the same individual cuckoo with this high error frequency in 2 consecutive years at the same site, so this syntax error was not temporally plastic. The relative rarity of such syntax errors means that repetitive redundancy (sensu^45,46^) in cuckoo calls effectively eliminates their functional effects. For these reasons, in acoustic signalling of cuckoos, we conclude that syntax errors do not appear to eliminate the functional effectiveness of the cuckoos’ intraspecific communication system.

## Methods

### Study area

The study was conducted in central Hungary, ca. 25–60 km south of Budapest, at around the settlements Alsónémedi (47°18′; 19°09′), Apaj (47°06′; 19°05′), Kunszentmiklós (47°01′; 19°07′) and Tass (47°01′; 19°01′) during the 2020 and 2021 breeding seasons. We also used heterospecific controls with Eurasian collared doves for comparisons conducted in the year 2016. In this study area common cuckoos can be found in high densities in their breeding season (May and June). They almost exclusively parasitize great reed warblers (*Acrocephalus arundinaceus*) locally, a large host which breeds in narrow reed-beds along small irrigation and flood-relief channels^47^.

All applicable international, national, and/or institutional guidelines for the care and use of animals were followed. Local animal ethics regulations and agreements were followed for fieldwork. All work complied with the Hungarian laws, and the Middle-Danube-Valley Inspectorate for Environmental Protection, Nature Conservation and Water Management, Budapest, provided permission for research (permit no. PE/KTF/17190-3/2015).

### Playback files

We used cuckoo calls recorded in May between 2016 and 2019. Recording were made with a Telinga Universal parabola dish, equipped with a Sennheiser ME-62 microphone, a K6 powering module, a FEL MX mono preamp, and a Marantz PMD-620 MKII recorder (sampling rate: 48 kHz, 24-bit quality)^30^.

We constructed ten different sound files for playback from the basic “cu-coo” calls:

#### Heterospecific (negative) control

(1) The calls of a neutral species from the local avifauna, the Eurasian collared dove, were used for interspecific vocalization control.

#### Natural (positive) control

(2) Normal (natural) “cu-coo” calls.

#### Experimental treatments; one-note calls

(3) Deleting the second note, i.e. contained “cu”, only.

(4) Deleting the first note, i.e. contained “coo”, only.

#### Two-note calls

(5) Reversal of the basic “cu-coo” call, i.e. “coo-cu”.

(6) Repeating the first note, and deleting the second note, i.e. “cu-cu”.

(7) Repeating the second note, and deleting the first note, i.e. “coo-coo”.

#### Three-note calls

(8) Repeating the first note, i.e. “cu-cu-coo”.

(9) Repeating the second note. i.e. “cu-coo-coo”.

#### Three-note natural

(10) Normal (but rare and context specific) “nat. cu-cu-coo”.

The experimental 3-note variant of the calls (“cu-cu-coo”; call type No. (8)) differs from our natural 3-note calls (“nat. cu-cu-coo”; call type No. (10)) in two out of the three acoustic parameters (length: F_1,18_ = 79.258, P < 0.001; maximum frequency: F_1,18_ = 58.243, P < 0.001) as an ANOVA revealed (Table 3). The natural variant has a shorter duration and higher maximum frequency than our experimental call type, which was constructed directly from the 2-note call by repeating the first element (Fig. 4). However, in the minimum frequency we did not find any deviation (F_1,18_ = 0.159, P = 0.695, Table 3).

Altogether 104 experimental trials were conducted and analyzed, 10–12 per trial types (see separate sample sizes per categories in Fig. 2, where we regarded the number of trials per playback type as sample size). Most of the trials (calls (5)–(10)) were played back in the 2021 breeding season, between May 11 and 26. Regarding the constraint in the size of the study area, the accessibility of birds, we also used trials of calls (2)–(4) played back between May 4 and 18, 2020, together with other categories not used in the present study^36^. Controls with collared dove calls were done in 2016 following the same protocols. Each playback file lasted 2 min, and it contained six calls for 10 s, repeated two times. This 30-s section was followed by a 15-s pause, and repeated two times (30 + 15 + 30 + 15 + 30 s), similarly to the file structure used by^30^. To reduce pseudo-replication (sensu^48,49^), in all types of playbacks (cu-coo, cu, coo, coo-cu, cu-cu, coo-coo, cu-cu-coo, cu-coo-coo, and dove control) we used each acoustic file for only one playback trial, except for one playback type. As the natural 3-note calls (“nat. cu-cu-coo”) typically overlap together with females’ bubbling calls (“duetting”^35^), we selected such calls without the bubbling calls, to broadcast clear signals. We had such natural “cu-cu-coo” calls non-overlapping with female bubble call from seven individuals, only. For this reason, in three cases we used two different call sequences from the same individuals in the “nat. cu-cu-coo” playback type.

All syntax error types generated here were heard and recorded by us at least once in the field (Fig. 1b,c), except the two-note monotypic calls (“cu-cu” and “coo-coo”).

### Field experiment

Playbacks were carried out under similar weather conditions (no rain and wind, between 7 and 11 h in the mornings). A playback site was selected where a male cuckoo was observed within 50 m, sitting on tree and calling. A JBL Xtreme 40 W loudspeaker was set on a tree about 1.5 m height, 20 m from the hide of the observer, and it was connected with an audio cable to a Lenovo TAB 2 A7 tablet, containing the playback files in wav 16-bit format. During playbacks cuckoos were followed continuously, and observations were dictated on a sound recorder (Tascam Dr05). We collected the following data: starting distance (the position of the focal bird from the speaker; m), the closest distance of the focal bird from the speaker during the 2-min playback (m), and latency (sec) as the time when the cuckoo started to approach the speaker during the playback. Cuckoos’ starting distances (the distance between cuckoo’s perch site and the loudspeaker) were measured with the help of a Bushnell Yardage Pro 800 rangefinder. Specifically, we estimated the closest distance between the flying cuckoos and the loudspeaker by sight, after personally having trained on visual assessment of distances with this rangefinder. We regarded an approach to the loudspeaker when the cuckoo left the tree where it was perched on at the start of a playback and flew toward the speaker. In contrast, when the focal bird changed position in the same tree at the starting point, or, rarely went into the other direction, these were not considered as approach movement. The next trial point was selected at a distance of about 500 m^36^, or more if the focal bird was continuously calling at the same site with known position, otherwise at least 1 km (c.f.^50^) to exclude the repeated use of the same individual cuckoo as a focal bird. The type of playback was drawn randomly at each site.

### Statistical analyses

We applied two fixed effect linear models when analysing the data. In the first model the response variable was reaction (Y/N), referring to whether or not the focal bird approached the speaker during the 2-min playback. In the model we used treatment as a predictor factor, and starting distance as a covariate. In the second model we replaced the binary response variable (Y/N) to closest distance (m) when cuckoos approached the speaker.

We used binary logistic regression for the comparison of male common cuckoos’ responses (Y/N; dependent variable) with the independent variables of 3-note playbacks (“cu-cu-coo” and “cu-coo-coo”), relative to natural 3-note cuckoo calls (“nat. cu-cu-coo”; reference category) and starting distance (m). For logistic regression the method “enter” was applied.

We ran one-way analysis of variance (ANOVA) to reveal if latencies to the different playback call types were different. As cuckoos showed no or almost no (0 ≤ n ≤ 2) response to a few playback categories (“coo”, “coo-coo”, “nat. cu-cu-coo”, and “dove control”), we excluded these categories from the comparison of latencies. For pairwise comparisons of cuckoos’ responses to different playback types we also used Tukey’s post hoc test after ANOVA. One-way ANOVA was also useful for the comparison of acoustic parameters of 3-note cuckoo calls, i.e. the natural “nat. cu-cu-coo”, experimental “cu-cu-coo” and “cu-coo-coo” (see above in “Methods”).

All statistical analyses were conducted using the SPSS ver. 17 package (SPSS Inc., Chicago, IL, USA).

## Data Availability

We uploaded representative sound files containing both calls with syntax errors and normal common cuckoo calls from our study site in Hungary to Xeno-Canto; codes XC661866 (containing a “cu” call; https://www.xeno-canto.org/661866), XC661875 (containing “cu-coo-coo” calls; https://www.xeno-canto.org/661875), and XC661876 (containing a “cu-coo-coo” call; https://www.xeno-canto.org/661876). This sound library also contains female bubbling calls together with male natural 3-note “cu-cu-coo” calls (XC422394, XC422426, and XC422443), and normal “cu-coo” calls of male cuckoos (XC323683, XC323807, XC323954, XC323955, XC380809, XC380974, XC380993, and XC381024) recorded at our study site. The analysed dataset is available through Figshare.com at https://figshare.com/s/14020d04cd4da869a4f3.
